# O5-7 Feasibility Study- A physiotherapy led remote ACHD Cardiac Rehabiliation Program

**DOI:** 10.1093/eurpub/ckac094.039

**Published:** 2022-08-29

**Authors:** Caroline Evans, Alan Graham Stuart

**Affiliations:** Cardiology, Bristol Heart Institute, Bristol, United Kingdom

**Keywords:** Remote congenital cardiac rehabilitation

## Abstract

**Problem:**

Prevalence of adult congenital heart disease (ACHD) is growing. Exercise is safe and effective at enhancing exercise capacity and quality of life (QOL) for ACHD patients but they are predominately sedentary with reduced exercise capacity and obese compared to peers. ACHD patients are concerned about their inactivity, however are uncertain of exercise suitability and safety and are unable to access specialist advice or cardiac rehabilitation (CR). Many ACHD patients are managed in specialist centres covering vast areas meaning classes are inappropriate. Acquired heart disease CR is cost-effective, improving QOL, exercise capacity, mortality and morbidity. ACHD patients potentially gain the same benefits. Remote cardiac rehabilitation (RCR) using technology could be a feasible model providing accessible specialist knowledge and individualised exercise prescription.

**Description of the problem:**

Cardiologists referred sedentary, complex ACHD patients for a 12-week RCR providing individualised exercise prescription, education and motivation via telephone clinics and free apps (PT Momentum, Pacer and Active 10). Initially, habitual exercise was encouraged, participants were coached to achieve the recommended UK PA guidelines. Self-efficacy for Exercise (SEE) and Satisfaction with Life Score (SWLS) were assessed comparing patients' responses upon program completion to baseline.

1. To determine the feasibility and acceptability of RCR to ACHD patients and quanitfy the impact of RCR on patient's physical activity (PA) and QOL.
2. To discover if technology supported programme can increase the PA level of ACHD patients.

**Results:**

From 23 referrals, 11 completed the program becoming more physically active, fulfilling the guidelines. Improvements were seen in both SEE (mean 30) and SWLS (mean 10). Patients deemed RCR an acceptable service delivery.

Participants (9) responded well to apps. Reasons for not using apps included data capacity (1) or no device (2).

**Lessons:**

ACHD patients engaged with RCR making it; viable, acceptable and practical solution improving PA levels and QOL.

RCR using app's is a feasible model however, alternatives are required.

**Main Messages:**

RCR enables ACHD patients to receive specialist knowledge, individualised, accessible and effective exercise prescription improving QOL and PA levels.

RCR potentially leads to substantial cost savings and improvements in morbidity and mortality of ACHD patients.

